# Association between plasma soluble P-selectin elements and progressive ischemic stroke

**DOI:** 10.3892/etm.2013.985

**Published:** 2013-02-28

**Authors:** QIAN WANG, WEILI ZHAO, SHUFENG BAI

**Affiliations:** 1 Department of Emergency, General Hospital of Chinese People’s Armed Police Forces, Beijing 100039;; 2 Department of Neurology, The First Affiliated Hospital of Henan University of Science and Technology, Luoyang 471003, P.R. China

**Keywords:** progressive cerebral infarction, P-selectin, risk factors

## Abstract

The aim of this study was to analyze the association between plasma-soluble P-selectin (sP-selectin) elements and progressive ischemic stroke (PIS) and to explore the pathogenesis of PIS. Patients with acute ischemic stroke who were admitted and hospitalized in the Department of Neurology between August 2010 and August 2011 were used as subjects in this study. The enrolled patients were divided into progressive (58 cases) and non-progressive groups (143 cases), based on changes in disease conditions. The normal control group included 40 cases. The sP-selectin levels and related risk factors of the three groups of patients were compared. sP-selectin levels in the progressive group showed the highest values on day 1 after progression and gradually decreased on days 3, 7 and 14. sP-selectin levels in the progressive and non-progressive groups on day 1 were higher compared with those in the control group (P<0.05) and the levels in the progressive group were higher compared with those in the non-progressive group (P<0.05). On days 3 and 7, levels in the progressive group were higher compared with those in the non-progressive group (P<0.05) and on day 14, levels in the progressive group remained higher compared with those in the non-progressive group (P>0.05). On days 1, 3 and 7, sP-selectin levels in the aortic atherosclerosis progressive group were higher compared with those in the aortic atherosclerosis non-progressive group (P<0.05), however on day 14, the difference between the two groups was not statistically significant (P>0.05). P-selectin levels had the most significant impact on the progressive group and the aortic atherosclerosis progressive group. P-selectin levels were high in patients with PIS and even higher in the aortic atherosclerosis progressive group and were closely correlated with the onset time of PIS.

## Introduction

Stroke is a common disease of the nervous system and ∼100 million individuals have an acute ischemic stroke each year in China. It is one of the diseases which are most likely to cause death in humans (1,2). Progressive ischemic stroke (PIS) (3) is a type of ischemic stroke which follows a clinical course where the primary nervous system symptoms and signs of ischemic stroke gradually progress or have a stepwise progressive course; disease progression generally occurs within 6 h to one week after onset. PIS has a poor prognosis and a high mortality rate, which is a burden for the families of patients and society, and it has therefore attracted increasing amounts of research attention. The progression of ischemic stroke, the mechanisms which induce progression and relevant risk factors have not been identified. Therefore, researching methods to implement early prediction, make a correct judgement on the condition and for timely and effective intervention to prevent the occurrence of ischemic stroke development has become the main focus in numerous studies.

There is no unanimous viewpoint with regard to the potential initiating factor for the progression of stroke. There are numerous studies on the causes, pathology and pathogenesis of PIS, however no consistent conclusion has been achieved. At present, stroke progression is considered to be a result of the joint interaction of a variety of pathogeneses and related factors. Platelet activation is important in the occurrence and development of stroke. There are numerous markers of platelet activation, one of which is P-selectin (CD62p), which has a high sensitivity and specificity. The level of CD62p reflects the degree of platelet activation and the status of platelet function. CD62p also mediates the platelet, neutrophil and endothelial cell adhesion function, thereby enhancing the reperfusion injury after stroke. The association between P-selectin and PIS is seldom reported.

In this study, plasma-soluble (sP)-selectin levels were determined for patients who were admitted within 24 h of the onset of an acute ischemic stroke. These were combined with relevant clinical data to investigate the association between P-selectin and PIS. We further explored the pathogenesis and etiology of PIS*,* with an aim to provide a reliable basis for targeted therapy in clinics and to lower the incidence of PIS, which is of clinical and social significance.

## Subjects and methods

### Case selection

Patients who had an acute ischemic stroke and were admitted and hospitalized in the Department of Neurology between August 2010 and August 2011 were used as subjects for this study. The patients in the control group were part of the normal population without any cerebrovascular diseases and received physical examination in the out-patient department of the hospital. This study was conducted in accordance with the Declaration of Helsinki and had approval from the Ethics Committee of Henan University of Science and Technology. Written informed consent was obtained from all participants.

Inclusion criteria were as follows: i) The patient should meet the criteria for ischemic stroke, as defined by the World Health Organization (4); ii) ischemic stroke was confirmed by a CT scan or magnetic resonance imaging (MRI); iii) the patient had consistent symptoms of neurological deficits; iv) the patient was admitted to hospital within 24 h of the onset of an acute ischemic stroke; v) the degree of clinical neurological deficits was scored using the U.S. National Institutes of Health Stroke Scale (NIHSS) score. Exclusion criteria were as follows: i) Acute ischemic cerebrovascular disease was present in combination with cerebral hemorrhage; ii) the patient had serious organ disease, inflammation, an autoimmune disease, cancer or a severe electrolyte imbalance; iii) the patient had a disturbance of consciousness at admission; iv) the patient had a disease of the hematopoietic or endocrine system or peripheral vascular disease; v) the patient had a cardiogenic cerebral embolism; vi) the patient was unable to participate in the relevant ancillary tests, including MRI, carotid ultrasonography and echocardiography.

The name, age, gender and history of hypertension, diabetes, smoking and drinking of the patients were registered in detail.

### Detection indexes

Detection indexes included blood routine, urine routine, stool routine, full sets of tests for blood clotting, full sets of tests for blood biochemistry, ECG and numerous others.

### Grouping

The NIHSS score was used to group the patients. The cases were scored using the NIHSS at admission and thereafter, the patients were scored at 8:30 every day until day 7. The scores for the degree of neurological deficits at the time when the condition had progressed to a peak were recorded. The scoring was conducted by two highly qualified neuro-physicians. According to the score for changes in condition (NIHSS), the patients were divided into the progressive and non-progressive groups.

Patients with a score change of ≥2 for neurological deficits following admission were included in the progressive group. There were 58 cases in the progressive group, including 34 males and 24 females with an average age of 63.19±10.23 years. Patients with a score change of <2 for neurological deficits following admission were included in the non-progressive group. There were 143 cases in the non-progressive group, including 82 males and 61 females with a mean age of 62.4±10.20 years. The control group included normal healthy subjects who received a physical examination in our hospital. There were 40 cases in the control group, including 23 males and 17 females with an average age of 60.7±10.26 years. The differences for age and gender between the three groups were not statistically significant (P>0.05).

Patients in the progressive and non-progressive groups were further divided into the aortic atherosclerosis and non-aortic atherosclerosis groups, according to whether they had the condition (5).

### Determination of P-selectin

Fasting blood samples of the patients in the progressive and non-progressive groups were collected on days 1, 3, 7 and 14; 2 ml of cubital venous blood was withdrawn in the mornings. Blood was collected once from the control group. The blood samples were placed in tubes containing 2% sodium citrate and whole samples were centrifuged at 3500 rpm for 10 min. The plasma was collected and preserved.

If the sample was not analyzed immediately, it was divided into two and placed in a −80°C refrigerator for preservation.

Determination of P-selectin was carried out using an Alisei microplate reader (Radim Company, Rome, Italy) and a human P-selectin kit, which was provided by Shanghai Yope Biotech Co. (Shanghai, China). The double-antibody sandwich method was adopted to determine the sP-selectin levels and the test was carried out according to the manufacturer’s instructions.

### Determination of blood-fat

Fasting venous blood samples (2 ml) were withdrawn on the morning after admission. Samples were placed in dry tubes and sent to the Clinical Laboratory of Biochemistry within the hospital. The blood-fat was detected by a kinase assay in 7600-020 fully automatic biochemistry analyzer (Hitachi Company, Tokyo, Japan). The reagent kits were provided by Shanghai Huifeng Medical Technology Co., Ltd. (Shanghai, China).

### Determination of high sensitivity C-reactive protein (hs-CRP)

Fasting venous blood samples (2 ml) were withdrawn on the morning after admission. Samples were placed in tubes containing a sodium heparin anticoagulant and sent to the Clinical Laboratory of Biochemistry within the hospital. The instrument used was a Hitachi 7600-020 and the reagent kit was produced by Shanghai Zhicheng Bioscience Co. (Shanghai, China). The determination was conducted according to the turbidimetric method.

### Statistical analysis

SPSS statistical software 13.0 was used for statistical analysis and measurement data are expressed as mean ± standard deviation. A single-factor analysis of variance was employed for comparison of data among the three groups, a t-test was used for comparison of data between two groups, a χ^2^ test was adopted for comparison of count data and binary logistic regression analysis was used for analysis of the risk factors. P<0.05 was considered to indicate a statistically significant difference.

## Results

### Analysis of the relevant factors

Age and smoking prevalence in the progressive and non-progressive groups were higher than in the control group (P>0.05 and P<0.05, respectively). With regard to gender, there was no significant difference between the progressive or non-progressive groups and the control group (P>0.05). The prevalence of hypertension, diabetes rate, blood lipid levels, hs-CRP levels and prevalence of a body temperature of >37.5°C were higher in the progressive and non-progressive groups than in the control group (P<0.05; Table I). The prevalence of hypertension, diabetes and a body temperature of >37.5°C was higher in the progressive group than in the non-progressive group (P<0.05). Blood lipid levels in the progressive group were higher than in the non-progressive group (P>0.05). Hs-CRP levels in the progressive group were higher than in the non-progressive group (P<0.01; Table I).

### Comparison of sP-selectin levels

sP-selectin levels in the progressive group exhibited the highest values on day 1 after progression and gradually decreased on days 3, 7 and 14. The sP-selectin levels in the progressive and non-progressive groups on day 1 were higher than in the control group (P<0.05) and the levels in the progressive group were higher than in the non-progressive group (P<0.05). On days 3 and 7, the levels in the progressive group were higher than in the non-progressive group (P<0.05) and on day 14, the levels in the progressive group remained higher than in the non-progressive group (P>0.05; Table II).

### Analysis of the relevant factors for patients with aortic atherosclerosis

The average age and lipid levels in the aortic atherosclerosis progressive group were higher than in the aortic atherosclerosis non-progressive group (P>0.05). With regard to gender, there was no significant difference between the aortic atherosclerosis progressive and non-progressive groups (P>0.05). The prevalence of hypertension, diabetes and a body temperature of >37.5°C was higher in the aortic atherosclerosis progressive group than in the aortic atherosclerosis non-progressive group (P<0.05; Table III).

### Comparison of sP-selectin levels for patients with aortic atherosclerosis

On days 1, 3 and 7, sP-selectin levels in the aortic atherosclerosis progressive group were higher than in the aortic atherosclerosis non-progressive group (P<0.05). However, on day 14, the difference between the two groups was not statistically significant (P>0.05; Table IV).

### Binary logistic regression analysis of multiple factors

The seven factors (history of hypertension, history of diabetes mellitus, a body temperature of >37.5°C, hs-CRP levels and P-selectin levels on days 1, 3 and 7) in the progressive and non-progressive groups which had significant differences were the independent variables and progression or non-progression following the occurrence of ischemic stroke was the dependent variable used to carry out binary logistic regression analysis. The four main factors which were entered into the regression model were a history of diabetes, history of hypertension and P-selectin levels on days 1 and 3. Results showed that P-selectin levels had the most significant impact on PIS (P-selectin levels on day 1: OR, 4.378; 95% CI, 1.217–15.972. P-selectin levels on day 3: OR, 3.019; 95% CI, 1.157–13.782. Table V).

### Binary logistic regression analysis of multiple factors for patients with aortic atherosclerosis

The six factors (history of hypertension, history of diabetes mellitus, a body temperature of >37.5°C and P-selectin levels on days 1, 3 and 7) in the aortic atherosclerosis progressive and non-progressive groups which had significant differences were the independent variables and progression (≥50%) or non-progression of atherosclerotic stenosis was the dependent variable used to carry out binary logistic regression analysis. The three main factors which were entered into the regression model were a history of diabetes and P-selectin levels on days 1 and 3. Results showed that P-selectin levels had the most significant impact on the aortic atherosclerosis progressive group (P-selectin levels on day 1: OR, 5.432; 95% CI, 1.319–17.893. P-selectin levels on day 3: OR, 5.017; 95% CI: 1.279–16.716. Table VI).

## Discussion

PIS is a unique subtype of stroke with a poor treatment efficacy, which is a burden to the families of patients and society, and as a result has attracted increasing amounts of research attention. The progression of stroke is considered to be a result of the joint interaction of a variety of pathogeneses and related factors. The risk factors and pathogenesis of PIS are unclear and these need to be further explored to allow the effective inhibition of its development; if achieved, this would be of clinical significance. Platelet activation is important in the occurrence and development of ischemic stroke.

Cell adhesion and agglomeration do not normally occur between platelets and endothelial cells. However, under pathological and physiological stimulation, such as when endothelial cells are damaged and subendothelial collagen is exposed, cell adhesion and agglomeration occurs to allow the platelets to be activated and form blood clots or to further develop the thrombus, thus causing ischemic stroke symptoms to become worse. Aortic atherosclerosis and unstable plaque and stenosis, in combination with the gradual increase of the thrombus size, are likely to gradually worsen the symptoms of neurological deficits; rupture or shedding of plaque may further worsen stroke symptoms. P-selectin, which is expressed by platelets in atherosclerosis, is essential to the effective interaction of monocytes and endothelial cells (6). Diabetes, hypertension, high cholesterol and hs-CRP are all risk factors of ischemic stroke. These may all directly or indirectly lead to the occurrence and development of ischemic stroke and the presence of these risk factors increase the chances of stroke progression. Although the presence of these factors may not definitely increase the progress of the stroke, timely intervention should be implemented for these risk factors to minimize the possibility of stroke progression. In this study, we explored the pathogenesis of PIS by investigating the variation of sP-selectin and relevant risk factors in PIS.

Platelet activation is important in PIS. P-selectin is a membrane glycoprotein, which is present in the platelet α-granule membrane and is also known as CD62p or platelet α-granule membrane protein-140. There are two types of CD62p, a surface membrane and a soluble type, and the main difference between the two types is that sP-selectin has a smaller molecular weight and lacks a transmembrane functional area. P-selectin is involved in physiological hemostasis and thrombosis. P-selectin-mediated platelets roll on the surface of endothelial cells to participate in hemostatic reactions (7). When endothelial cells in the early stage of the vein are activated, CD62p migrates to the surface of the endothelial cells. The action of the glycoprotein ligand of P-selectin in micro-bubbles causes the expression of tissue factors to be increased, thereby mediating the initiation of the endogenous coagulation process (8). P-selectin mediates the adhesion of leukocytes and endothelial cells. When P-selectin combines with corresponding ligands on the surface of leukocytes, it causes the slow rolling of leukocytes, which ultimately causes an increased concentration of white blood cells in inflamed areas (9). Studies using P-selectin-deficient rats revealed a serious disorder of leukocyte adhesion function in these rats, suggesting that CD62p has a role in white blood cells to mediate leukocyte activation and adhesion and to release inflammatory substances (10). P-selectin aids the reperfusion of injuries following ischemia and promotes the formation of atherosclerosis. André *et al*(11) showed that CD62p expressed in platelets was able to signal monocytes to aggregate at the thrombus site and these particles are an important source of tissue factors in the thrombus plaque component.

Numerous studies have investigated sP-selectin. A study by Kozuka *et al* showed that soluble P- and E-selectin levels were significantly higher in ischemic stroke patients than in control patients. P-selectin levels continued to rise until the sub-acute phase, while in patients with lacunar infarction, soluble E-selectin levels were higher only during the acute phase (12). Studies have shown that the expression of CD62p and CD63 in patients with ischemic stroke was higher than in control patients on days 1, 3 and 7 following the onset of the disease. This difference was statistically significant, which indicates that platelet activation was clearly increased in the acute phase of ischemic stroke (13). Patrik *et al* investigated blood CD62p expression within 24 h and 3 months after onset in 135 patients with transient ischemic attack (TIA) and complete ischemic stroke. CD62p levels in the two groups of patients were significantly increased within 24 h, as compared with that of a healthy control group. When the two stroke groups were compared with each other, there were no significant differences in CD62p levels and levels were recovered to normal three months later, suggesting that there was not a clear correlation between platelet activation and stroke size (14). A study by Marquardt *et al* on patients with ischemic stroke, which featured a healthy control group and a stroke group, showed that CD62p and CD63 expression on the first day following stroke onset was higher than in healthy control patients. CD63 expression continued to rise until day 90 after onset of the disease, whereas CD62p expression rapidly decreased (15).

In the present study, plasma CD62p levels were increased in patients with progressive stroke, however, this decreased over time. In the analysis of relevant risk factors, hypertension, diabetes, a body temperature of >37.5°C and hs-CRP were correlated with progressive stroke. Binary logistic regression analysis was conducted on these significant factors and the results showed that P-selectin levels had the most significant impact on PIS.

Binary logistic regression analysis was carried out on the five factors which had significant differences for the aortic atherosclerosis progressive and non-progressive groups and the results showed that P-selectin levels had the most significant impact on the aortic atherosclerosis progressive group. In this study, plasma CD62p levels in the progressive and aortic atherosclerosis progressive groups were high, indicating that platelets were involved in the occurrence and development of ischemic stroke and the formation of atherosclerosis. Furthermore the significant increase in CD62p expression may be important in the pathogenesis of PIS.

PIS occurs as a result of the joint effect of a variety of pathogeneses and multiple risk factors. In this study, the univariate analysis of relevant factors in PIS revealed that hypertension, diabetes, a body temperature of >37.5°C, plasma hs-CRP and blood-fat in the progressive group were higher than in the non-progressive and control groups and these results had statistical significance.

There are numerous studies on the correlation between hypertension and progressive stroke and it is one of the risk factors for cerebrovascular diseases. Long-term hypertension may cause atherosclerosis, which leads to cerebral artery stenosis (16). Long-term hypertension leads to a reduction in cerebral blood in the ischemic region, the occlusion of large vessels and macrovascular disease in patients. This causes a decrease in blood flow at distal end of local stenosis, ischemia to occur in the sites where collateral circulation was poor, aggravation of the degree of stenosis and the occlusion of blood vessels, which may further develop into PIS. In patients with hypertension, the automatic adjustment range of cerebral blood flow is minor and the baseline arterial blood pressure is high. Even a slight decline in blood pressure is able to cause a decrease in cerebral blood flow, which causes further ischemia in the penumbra region, thus leading to stroke progression. Clinical studies (17) showed that stroke patients with hypertension had a higher mortality rate and high blood pressure was closely correlated with the deterioration of nerve function. The results of the present study demonstrated that the incidence of hypertension in the progressive group was higher than in the non-progressive and control groups, indicating that a history of hypertension is correlated with progressive stroke, which is consistent with the results of previous relevant studies.

There have been numerous studies by domestic and foreign researchers with regard to the correlation between diabetes and the progression of ischemic stroke. High glucose levels in the blood of patients with diabetes causes a glucose increase in brain tissue, an increase in anaerobic glycolysis and an increase in brain lactate. The accumulation of lactic acid aggravates acidosis and may cause intracellular poisoning and increase ischemic necrosis. Hyperglycemia may promote the oxidation of cells and damage endothelial cells, causing damage to the vessel wall and aggravating cerebral infarction symptoms. It may also lead to mitochondrial damage and, ultimately, cell death. Clinical trials by Caplan (18) showed that diabetes led to a 1.9 times increased risk for the progression of ischemic stroke.

Relevant foreign studies (19) have shown that the probability of having early progression and the mortality rate was high in diabetic patients with ischemic stroke, indicating that glucose levels affect the occurrence and development of stroke. High glucose levels in diabetics leads to the synthesis and release of plasminogen activator inhibitor-l by inhibition of tissue plasminogen activator, causing inhibition of the fibrinolytic system, which leads to the occurrence and development of ischemic stroke (20). A clinical study by Lloyd-Jones *et al*(21), which used diffusion-weighted imaging (DWI) in patients with ischemic stroke, showed that patients with acute ischemic stroke who were admitted to hospital showed persistent high blood glucose, expanded lesions and/or a relatively high poor prognosis rate. The present study showed that increased blood glucose was a risk factor for acute ischemic stroke progression. The incidence of diabetes in the progressive group was higher than in the non-progressive and control groups, indicating that there is a correlation between diabetes and progressive stroke, which is consistent with the results of previous domestic and international studies. This suggests that the blood glucose of patients with ischemic stroke should be timely monitored in the acute phase and reasonable measures for decreasing blood glucose should be taken.

An increased body temperature in stroke patients aggravates the clinical symptoms of ischemic stroke, increases cerebral metabolism, affects prognosis and may even be fatal. In this experiment, the prevalence of a body temperature of >37.5°C was higher in the progressive group than in the non-progressive group, indicating that an elevated body temperature is a risk factor for progressive stroke and therefore the cooling of body temperature should be applied based on the individual condition of the patient and if body temperature is elevated.

hs-CRP is a protein which is synthesized in the liver and is a sensitive inflammatory marker. Ridker *et al*(22) showed that, among the predictors of cerebrovascular events, hs-CRP has better predictability compared with other indicators. A study on elderly PIS patients with a high proportion of obesity and Parkinson’s disease demonstrated that, the mortality rate in the first month was as high as 31% (23).

In conclusion, P-selectin level was high in patients with PIS and even higher in patients with progressive aortic atherosclerosis, and was closely correlated to the onset time of PIS. Detection of this index has important clinical significance for intervention and treatment of PIS.

## Figures and Tables

**Table I:** Analysis of relevant factors for the progressive, non-progressive and control groups.

Factors	Progressive group (58 cases)	Non-progressive group (143 cases)	Control group (40 cases)
Average age (years), mean ± SD	63.19±10.23	62.4±10.20	60.7±10.26
Gender (male/female)	34/24	82/61	23/17
Smoking history (%)	42.97	51	20
Hypertension (%)	90.12	52.05	0
Diabetes (%)	68.98	26.79	0
Total cholesterol (mmol/l), mean ± SD	5.40±1.04	5.19±1.01	4.03±0.68
Triglyceride (mmol/l), mean ± SD	2.78±2.68	2.01±0.92	1.35±0.38
Fasting blood-glucose (mmol/l), mean ± SD	8.03±2.57	5.54±1.47	5.0±1.1
Low density lipoprotein (mmol/l), mean ± SD	1.36±0.27	1.32±0.19	0.97±0.48
Hs-CRP (mg/l), mean ± SD	7.64±3.51	5.85±2.14	3.51±1.25
Body temperature >37.5°C (%)	33.61	10.10	0

**Table II:** Comparison of P-selectin levels (*μ*g/l) between the progressive, non-progressive and control groups (mean ± SD).

Day	Progressive group	Non-progressive group	Control group
1	27.01±2.56	20.15±3.01	8.27±3.96
3	25.4±3.21	18.2±3.89	
7	21.7±3.07	15.4±3.68	
14	11.08±2.89	10.06±2.86	

**Table III:** Comparison of relevant factors between two groups.

Factors	Large-artery atherosclerosis progressive group (38 cases)	Large-artery atherosclerosis non-progressive group (52 cases)	P-value
Male (%)	61	60	0.154
Age (years), mean ± SD	64.4±10.7	63.1±10.56	0.072
Hypertension (%)	95	60	0.038
Diabetes (%)	74	32	0.043
Smoking history (%)	56.8	60	0.074
Total cholesterol (mmol/l), mean ± SD	5.60±1.04	5.20±1.01	0.063
Low density lipoprotein (mmol/l), mean ± SD	1.47±0.27	1.33±0.19	0.547
Triglyceride (mmol/l), mean ± SD	2.78±2.68	1.98±0.92	0.108
Body temperature >37.5°C (%)	40.72	15.57	0.021

**Table IV:** Comparison of P-selectin levels (*μ*g/l) between two groups.

Day	Large-artery atherosclerosis progressive group (38 cases)	Large-artery atherosclerosis non-progressive group (52 cases)
1	32.4±2.78	24.1±1.19
3	30.6±4.46	22.6±4.50
7	26.7±5.79	19.8±5.68
14	12.42±3.89	11.65±4.41

**Table V:** Binary logistic regression analysis between progressive group and non-progressive group.

Factors	B	SE	Wald	OR	P-value	95% CI
History of diabetes (%)	0.857	0.418	4.014	2.351	0.043	1.021–5.355
Hypertension (%)	0.914	0.567	4.079	2.567	0.027	1.075–7.859
CD62p content on day 1 (*μ*g/l)	1.607	0.756	5.019	4.378	0.025	1.217–15.972
CD62p content on day 3 (*μ*g/l)	1.342	0.679	4.378	3.019	0.023	1.157–13.782
Constant	−3.791	1.237	9.978	0.034	0.001	

B, regression coefficient; SE, standard error; Wald, Wald P-value; OR, odds ratio; CI, confidence interval; CD62p, P-selectin.

**Table VI:** Binary logistic regression analysis between the large-artery atherosclerosis progressive and non-progressive groups.

Factors	B	SE	Wald	OR	P-value	95% CI
History of diabetes (%)	0.582	0.489	3.985	2.486	0.047	1.103–5.576
Hypertension (%)	1.705	0.764	6.023	5.432	0.024	1.319–17.893
CD62p content on day 1 (*μ*g/l)	1.534	0.583	4.978	5.017	0.021	1.279–16.716
CD62p content on day 3 (*μ*g/l)	−3.549	1.156	10.954	0.046	0.001	

B, regression coefficient; SE, standard error; Wald, Wald P-value; OR, odds ratio; CI, confidence interval; CD62p, P-selectin.
